# Physician Coaching by Professionally Trained Peers for Burnout and Well-Being: A Randomized Clinical Trial

**DOI:** 10.1001/jamanetworkopen.2024.5645

**Published:** 2024-04-12

**Authors:** Stephanie B. Kiser, J. David Sterns, Po Ying Lai, Nora K. Horick, Kerri Palamara

**Affiliations:** 1 Division of Palliative Care and Geriatric Medicine, Department of Medicine, Massachusetts General Hospital, Boston; 2 Department of Occupational Medicine, Naval Medical Readiness and Training Command New England, US Navy, Portsmouth, New Hampshire; 3 Department of Biostatistics, Massachusetts General Hospital, Boston; 4 Center for Physician Well-Being, Department of Medicine, Massachusetts General Hospital, Boston

## Abstract

**Importance:**

Physician burnout is problematic despite existing interventions. More evidence-based approaches are needed.

**Objective:**

To explore the effect of individualized coaching by professionally trained peers on burnout and well-being in physicians.

**Design, Setting, and Participants:**

This randomized clinical trial involved Mass General Physician Organization physicians who volunteered for coaching from August 5 through December 1, 2021. The data analysis was performed from February through October 2022.

**Interventions:**

Participants were randomized to 6 coaching sessions facilitated by a peer coach over 3 months or a control condition using standard institutional resources for burnout and wellness.

**Main Outcomes and Measures:**

The primary outcome was burnout as measured by the Stanford Professional Fulfillment Index. Secondary outcomes included professional fulfillment, effect of work on personal relationships, quality of life, work engagement, and self-valuation. Analysis was performed on a modified intention-to-treat basis.

**Results:**

Of 138 physicians enrolled, 67 were randomly allocated to the coaching intervention and 71 to the control group. Most participants were aged 31 to 60 years (128 [93.0%]), women (109 [79.0%]), married (108 [78.3%]), and in their early to mid career (mean [SD], 12.0 [9.7] years in practice); 39 (28.3%) were Asian, 3 (<0.1%) were Black, 9 (<0.1%) were Hispanic, 93 were (67.4%) White, and 6 (<0.1%) were of other race or ethnicity. In the intervention group, 52 participants underwent coaching and were included in the analysis. Statistically significant improvements in burnout, interpersonal disengagement, professional fulfillment, and work engagement were observed after 3 months of coaching compared with no intervention. Mean scores for interpersonal disengagement decreased by 30.1% in the intervention group and increased by 4.1% in the control group (absolute difference, −0.94 poimys [95% CI, −1.48 to −0.41 points; *P* = .001), while mean scores for overall burnout decreased by 21.6% in the intervention group and increased by 2.5% in the control group (absolute difference, −0.79 points; 95% CI, −1.27 to −0.32 points; *P* = .001). Professional fulfillment increased by 10.7% in the intervention group compared with no change in the control group (absolute difference, 0.59 points; 95% CI, 0.01-1.16 points; *P* = .046). Work engagement increased by 6.3% in the intervention group and decreased by 2.2% in the control group (absolute difference, 0.33 points; 95% CI, 0.02-0.65 points; *P* = .04). Self-valuation increased in both groups, but not significantly.

**Conclusions and Relevance:**

The findings of this hospital-sponsored program show that individualized coaching by professionally trained peers is an effective strategy for reducing physician burnout and interpersonal disengagement while improving their professional fulfillment and work engagement.

**Trial Registration:**

ClinicalTrials.gov Identifier: NCT05036993

## Key Points

**Question:**

Does coaching by professionally trained physician peers reduce burnout and improve well-being, workplace satisfaction, and engagement for physicians?

**Findings:**

In this randomized clinical trial of 138 physicians, participants who received 3 months of coaching by professionally trained physician peers had a statistically significant reduction in interpersonal disengagement and burnout, with improvement in professional fulfillment and work engagement.

**Meaning:**

These findings show that physician peer coaching is an effective strategy for reducing burnout and improving well-being.

## Teaser

This randomized clinical trial explores the effect of individualized coaching by professionally trained peers on physician burnout and well-being.

## Introduction

Physician burnout is an epidemic characterized by emotional exhaustion, depersonalization, and sense of low personal accomplishment from chronic workplace stress.^1^ Prevalence is estimated at 30% to 63% of physicians experiencing symptoms of burnout at any time, including feelings of depletion, cynicism, and reduced professional efficacy.^2,3,4^ Burnout is associated with poor-quality care, increased self-reported medical errors, and decreased efficiency.^5,6^ The individual consequences include increased risk of substance use, mood disorders, and suicidal ideation.^7,8,9,10^ Systemic problems exacerbated by the COVID-19 pandemic have created profound emotional, psychological, and moral distress in clinicians and increased burnout.^11,12,13^

Contributors to physician burnout include work factors such as inefficient work processes, such as electronic health record documentation and high workloads; personal characteristics, such as engaging in unhelpful coping strategies and inadequate support systems; and organizational factors, such as negative leadership behavior and lack of opportunities.^14,15^ Addressing burnout requires evidence-based approaches targeting the individual and systems levels while also considering resource limitations.^16^

Coaching involves partnering with individuals to maximize personal and professional potential, yet it can also be applied at the systems level.^17^ Distinct from mentoring and counseling, coaching focuses on self-developed, goal-oriented change. Coaching has led to improved performance and skills, well-being, and coping in non–health care settings.^18^ Findings from an initial study of physician coaching support coaching’s potential as a strategy for addressing burnout and improving well-being by decreasing emotional exhaustion, improving quality of life, and fostering resilience.^19^

Prior coaching studies have presented implementation and access limitations, including a reliance on external coaching resources or a small number of internally trained coaches.^19,20,21^ Our study takes a unique approach by supporting the training and certification of physician peers internal to our organization to provide coaching in order to decrease resource requirements and improve accessibility. We hypothesized that peer-to-peer individualized professional coaching would lead to improved physician well-being, workplace satisfaction, engagement, and burnout.

## Methods

### Study Design, Setting, and Participants

We conducted a single-site, randomized clinical trial across the Massachusetts General Physicians Organization from August 5 through December 1, 2021. The study was approved by the Mass General Brigham institutional review board and followed the Consolidated Standards of Reporting Trials (CONSORT) reporting guideline. The trial protocol with plan for statistical analysis and the detailed institutional review board protocol are provided in Supplement 1. A recruitment goal of 100 physicians was chosen to provide 80% power to detect a 0.3- to 0.5-SD minimally important difference effect size in our primary outcome.^22^ Study participants were recruited through 2 email announcements with embedded links to provide informed consent and complete a baseline well-being assessment via REDCap software (Vanderbilt University). Any Massachusetts General Physicians Organization practicing physician who completed the baseline assessment was eligible. Of the 3247 physicians who received the email announcement, interested participants were enrolled on a first-come, first-served basis until reaching a recruitment maximum of 138, the limit of coach availability.

### Allocation and Follow-Up

Study participants were randomized to a coaching group and a delayed intervention group, which served as a control arm for the first 3 months, using a REDCap-generated allocation algorithm in a 1:1 ratio stratified by gender (woman, man, nonbinary, or prefer not to say) and department of medicine (yes or no). Participants completed an initial baseline assessment and 3-month assessment. Incentive items (journal and cell phone charger) were sent to participants who completed the survey.

### Intervention

Participants randomized to the coaching intervention self-selected their coach from a signup platform that contained coach biographies, headshots, and availability. Pairs engaged in a 1.5-hour initial coaching session followed by 5 additional 60-minute sessions, for a total of 6 coaching sessions over a 3-month period. Coaches were physician peers certified by Wellcoaches, a coach training company endorsed by the American College of Sports Medicine and the American College of Lifestyle Medicine.^23^ Coaches received a $2500 stipend for time spent in training and certification, and $200 per hour for coaching. Physician coaches and study participants were expected to maintain their clinical responsibilities throughout the study. Coaching was provided over the phone or through videoconferencing. During the initial session, the coach facilitated introductions, rapport building, and expectation setting, as well as the creation of a coaching vision and 3-month and weekly goals. The focus for follow-up sessions was individualized to the needs and desired growth areas of the study participant but generally followed the framework of a check-in, goal review, generative moment, new goal setting, and session conclusion. Participants randomized to the control group were sent wellness resources but received no additional intervention during the 3-month study period.

### Outcome Measures

Baseline and 3-month assessments included validated instruments measuring the outcomes of interest and several items assessing fidelity of the coaching intervention. The outcomes of interest were burnout, professional fulfillment, work engagement, self-valuation, quality of life, and effect of work on personal relationships.

#### Burnout

Burnout was measured through a modified Maslach Burnout Inventory (mMBI) and the work exhaustion and interpersonal disengagement subscales of the Stanford Professional Fulfillment Index (PFI).^1,24,25^ The mMBI consists of 2 single-item measures of emotional exhaustion (“I feel burned out from work”) and depersonalization (“I have become more callous toward people since I took this job”) from the 22-item MBI, measured on a 7-point scale (range, 0-6 points, with higher scores indicating greater burnout). The mMBI has provided consistent stratification of burnout risk among physicians comparable to the full assessment.^1,24^ The PFI consists of 16 items measured on a 5-point Likert scale, which assess work exhaustion (items 7-10) and interpersonal disengagement (items 11-16) similarly to the mMBI. Mean scores from the work exhaustion and interpersonal disengagement subscales are averaged and then multiplied by 2.5 for an overall burnout score (range, 0-10 points), with higher scores being sensitive and specific in identifying physicians experiencing higher levels of burnout.^25,26^

#### Professional Fulfillment, Work Engagement, and Self-Valuation

Professional fulfillment, work engagement, and self-valuation were measured by the professional fulfillment subscale of the PFI, the Utrecht Work Engagement Scale-9 (UWES-9), and the Self-Valuation Scale. The professional fulfillment subscale (items 0-6) assesses intrinsic reward derived from work, with cutoff scores greater than 7.5 points being sensitive and specific for a higher self-reported quality of life among physicians.^25^ The Utrecht Work Engagement Scale-9 contains 9 items scored on a 7-point scale assessing personal identification with one’s work, with higher scores indicating greater work engagement.^27^ The Self-Valuation Scale measures the degree to which physicians can care for themselves within their practice through 4 items on a 5-point Likert scale. Overall scores range from 0 to 16 points, with scores of 8 points or less being strongly associated with burnout and sleep-related impairment.^28^

#### Quality of Life and Effect of Work on Personal Relationships

The Quality of Life Scale and Impact of Work on Personal Relationships Scale were also administered. The Quality of Life Scale asks respondents to rate their quality of life from “as bad as it can be” (score of 0) to “as good as it can be” (score of 100) on a previously validated visual analog scale.^29^ The Impact of Work on Personal Relationships Scale consists of 4 items measured on a 5-point Likert scale, with higher scores indicating a more negative effect of work on personal relationships over the past year.^30^

#### Fidelity and Program Satisfaction

Participants were asked to rate quality of communication, whether a coaching vision and goals were established, themes addressed, and whether coaches primarily gave advice or guided the discussion, as markers of intervention fidelity.^19,31^ Finally, participants were asked whether they would consider coaching again in the future as a marker of program satisfaction.

### Statistical Analysis

The data analysis was performed from February through October 2022. Primary quantitative analysis was performed on a modified intention-to-treat basis using linear and logistic mixed-effects regression models to estimate the mean scores or proportions at each study time point. Participants who did not receive coaching after randomization and were lost to follow-up were excluded from the analysis. Models included as covariates the participant’s gender, department, and a random participant effect to account for the correlation among repeated assessments provided by each participant at multiple time points. Race and ethnicity were self-identified and measured to compare with our institution’s demographics to understand if there is inequity in accessing services. Contrasts were used to estimate and compare the change in means or proportions from baseline to 3 months between study groups. Percent change was calculated by dividing model-based estimates of the within-group change by the baseline mean or proportion. A 2-sided *P* ≤ .05 was considered statistically significant by linear mixed-effects models for all analyses without correction for multiple comparisons. Analyses were conducted using SAS, version 9.4 software (SAS Institute Inc).

## Results

Of the 138 physicians who provided informed consent and completed a baseline assessment, 67 were randomly allocated to the coaching intervention group and 71 to the control group (Figure). The baseline characteristics of participants in both groups were similar with respect to gender, age, clinical role, and a history of prior coaching (Table 1). Most participants were aged 31 to 60 years (128 [93.0%]), and the majority of participants in both groups were women (109 [79.0%] compared with 29 men [21.0%]; 2 preferred not to say), White (93 [67.4%] compared with 39 Asian [28.3%]; 3 Black [<0.1%], 9 Hispanic [<0.1%], and 6 [<0.1%] other race and ethnicity), married (108 [78.3%]), and in their early to mid career (mean [SD] of 12.0 [9.7] years in practice). The intervention group had a higher number of participants identifying as Asian (22 [32.8%] vs 17 [23.9%]) and divorced or separated (7 [10.4%] vs 2 [2.8%]) and a lower number of participants having primary caregiving responsibilities (49 [73.1%] vs 59 [83.1%]). Intervention group participants had a longer mean (SD) time in practice of 12.8 (8.9) years vs 11.7 (10.0) years in the control group. Most participants were either at the instructor (57 [46.3%]) or assistant professor (48 [39.0%]) levels. There were no statistically significant differences in baseline assessments of burnout, professional fulfillment, work engagement, effect of work on personal relationships, self-valuation, or quality of life between the intervention and control groups (Table 2).

**Figure.**
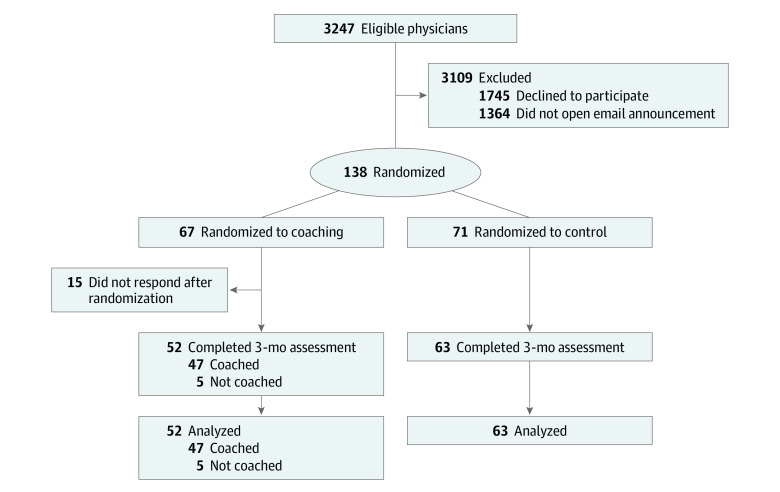
Study Flow Diagram

**Table 1.** Baseline Demographic Characteristics of Randomized Participants^a^

Characteristic	Randomized to intervention (n = 67)	Received coaching intervention (n = 52)	Control (n = 71)
Gender			
Woman	52 (77.6)	40 (76.9)	57 (80.3)
Man	14 (20.9)	10 (19.2)	13 (18.3)
Nonbinary	0	0	0
Prefer not to say	1 (1.5)	1 (1.9)	1 (1.4)
Race and ethnicity			
American Indian or Alaska Native	0	0	0
Asian	22 (32.8)	15 (28.8)	17 (23.9)
Black	2 (3.0)	1 (1.9)	1 (1.4)
Hispanic, Latinx, or of Spanish origin	6 (9.0)	5 (9.6)	3 (4.2)
Native Hawaiian or Pacific Islander	0	0	0
White	40 (59.7)	33 (63.7)	53 (74.6)
Other^b^	4 (6.0)	3 (5.8)	2 (2.8)
Age group, y			
<31	0	0	0
31-40	28 (41.8)	19 (36.5)	28 (39.4)
41-50	24 (35.8)	21 (40.4)	29 (40.8)
51-60	10 (14.9)	9 (17.3)	9 (12.7)
>60	5 (7.5)	3 (5.8)	5 (7.0)
Current relationship status			
Single	9 (13.4)	5 (9.6)	5 (7.0)
Married	50 (74.6)	40 (76.9)	58 (81.7)
Widowed	0	0	0
Divorced or separated	7 (10.4)	7 (13.5)	2 (2.8)
Partner	1 (1.5)	0	6 (8.5)
Employment			
Full time	61 (91.0)	46 (88.5)	61 (86.0)
Part time	6 (9.0)	6 (11.5)	10 (14.1)
Primary clinical role			
Procedural	20 (29.9)	13 (25.0)	20 (28.2)
Nonprocedural	47 (70.1)	39 (75.0)	51 (71.8)
Department of medicine			
Yes	24 (35.8)	17 (32.7)	24 (33.8)
No	43 (64.2)	35 (67.3)	47 (66.2)
Faculty appointment			
Instructor	31 (46.3)	21 (40.4)	36 (50.7)
Assistant professor	22 (32.8)	19 (36.5)	29 (40.8)
Associate professor	9 (13.4)	9 (17.3)	5 (7.0)
Full professor	5 (7.5)	3 (5.8)	1 (1.4)
No. of years in practice, mean (SD)	12.4 (9.2)	12.8 (8.9)	11.7 (10.0)
Caregiving responsibilities outside of work	49 (73.1)	40 (76.9)	59 (83.1)
Prior history of certified coaching	13 (19.4)	9 (17.3)	18 (25.4)

^a^ Data are presented as No. (%) unless otherwise specified.

^b^ No further data were collected if participants chose other.

**Table 2.** Baseline Burnout, Professional Fulfillment, Work Engagement, and Quality-of-Life Characteristics of Randomized Study Groups^a^

Characteristic	Randomized to intervention (n = 67)	Received coaching intervention (n = 52)	Control (n = 71)
Burnout^b^			
Work exhaustion (PFI subscore)	4.28 (1.76)	4.10 (1.73)	4.39 (2.15)
Interpersonal disengagement (PFI subscore)	2.77 (1.73)	2.73 (1.88)	2.90 (1.88)
Overall burnout (PFI WE + ID score)	3.37 (1.52)	3.28 (1.58)	3.50 (1.83)
Emotional exhaustion burnout (mMBI subscore)	3.31 (1.61)	3.23 (1.59)	3.35 (1.68)
Depersonalization burnout (mMBI subscore)	1.87 (1.61)	1.85 (1.67)	2.18 (1.73)
Engagement and quality of life			
Professional fulfillment (PFI subscore)^c^	5.55 (1.89)	5.47 (1.99)	5.87 (1.99)
Work engagement (UWES-9 score)^d^	3.98 (1.20)	3.90 (1.26)	4.03 (1.28)
Negative effect of work on personal relationships (IWPR score)^e^	3.92 (2.52)	3.56 (2.58)	3.94 (2.71)
Self-valuation (Self-Valuation Scale score)^f^	6.69 (2.87)	7.02 (2.91)	7.07 (3.91)
Quality of life^g^	59.75 (18.49)	61.50 (16.65)	60.54 (20.85)

Abbreviations: ID, interpersonal disengagement; IWPR, Impact of Work on Personal Relationship Scale; mMBI, modified Maslach Burnout Inventory; PFI, Stanford Professional Fulfillment Index; UWES-9, Utrecht Work Engagement Scale-9; WE, work exhaustion.

^a^ Data are presented as mean (SD) points unless otherwise specified.

^b^ Range of 0 to 10 points, with higher scores sensitive and specific in identifying physicians experiencing higher levels of burnout.

^c^ Cutoff scores greater than 7.5 points indicate sensitivity and specificity for a higher self-reported quality of life.

^d^ Higher scores on a 7-point scale indicate greater work engagement.

^e^ Higher scores on a 5-point Likert scale indicate a more negative effect of work on personal relationships over the past year.

^f^ Overall scores range from 0 to 16 points, with scores of 8 points or less strongly associated with burnout and sleep-related impairment.

^g^ Range of 0 (as bad as it can be) to 100 (as good as it can be) on a visual analog scale.

Of the 67 physicians randomized to coaching, 52 (77.6%) were matched with a coach and completed at least 1 coaching session. The remaining 15 physicians (22.4%) were lost to follow-up after completion of their baseline survey and randomization. Our modified intention-to-treat analyses included the 71 participants randomized to the control arm and the 52 participants who were randomized to the intervention arm and completed at least 1 coaching session . A total of 110 of these 123 physicians (89.4%) completed the 3-month survey (intervention, 47 of 52 [90.4%]; control, 63 of 71 [88.7%]). Physicians who received coaching engaged in a mean (SD) of 5.6 (1.3) sessions per participant.

Changes from baseline to 3 months post intervention in the intervention and control groups are described in Table 3. Physicians who received coaching had a 21.6% reduction in mean PFI burnout score compared with a 2.5% increase in the control group (absolute difference, −0.79 points; 95% CI, −1.27 to −0.32 points; *P* = .001). There was a statistically significant difference in the interpersonal disengagement subscale score (intervention vs control, 30.1% vs 4.1%; absolute difference, −0.94 points [95% CI, −1.48 to −0.41 points; *P* = .001]); there was no difference in the work exhaustion subscale score (intervention vs control, −12.4% vs 0.2%; absolute difference, −0.52 points [95% CI, −1.19 to 0.15 points; *P* = .13]). There were no statistically significant differences in emotional exhaustion or depersonalization using the mMBI.

**Table 3.** Changes From Baseline to 3 Months Post Intervention

Characteristic	Intervention group (n = 52)		Control group (n = 63)		Absolute difference, intervention vs control (95% CI), points	*P* value
	Absolute change, mean (95% CI), points	Relative change, %	Absolute change, mean (95% CI), points	Relative change, %		
Burnout						
Work exhaustion	−0.51 (−1.02 to 0.00)	−0.12	0.01 (−0.43 to 0.45)	0.00	−0.52 (−1.19 to 0.15)	.13
Interpersonal disengagement	−0.82 (−1.23 to −0.42)	−0.30	0.12 (−0.23 to 0.47)	0.04	−0.94 (−1.48 to −0.41)	.001
Overall burnout	−0.71 (−1.06 to −0.35)	−0.22	0.09 (−0.22 to 0.40)	0.03	−0.79 (−1.27 to −0.32)	.001
Emotional exhaustion	−0.12 (−0.51 to 0.26)	−0.04	−0.04 (−0.37 to 0.30)	−0.01	−0.09 (−0.60 to 0.42)	.73
Depersonalization	−0.2 (−0.6 to 0.2)	−0.1	0.1 (−0.2 to 0.5)	0.1	−0.4 (−0.9 to 0.2)	.18
Engagement and quality of life						
Professional fulfillment	0.59 (0.15 to 1.02)	0.11	0.00 (−0.38 to 0.38)	0.00	0.59 (0.01 to 1.16)	.046
Work engagement	0.25 (0.01 to 0.48)	0.06	−0.09 (−0.29 to 0.12)	−0.02	0.33 (0.02 to 0.65)	.04
Effect of work on personal relationships	0.13 (−0.50 to 0.77)	0.04	–0.28 (−0.83 to 0.28)	−0.07	0.41 (−0.43 to 1.25)	.34
Self-valuation	1.40 (0.62 to 2.18)	0.20	0.40 (−0.28 to 1.08)	0.06	1.00 (−0.04 to 2.04)	.06
Quality of life	2.35 (−2.92 to 7.61)	0.04	−0.21 (−4.77 to 4.35)	0.00	2.56 (−4.41 to 9.52)	.47

Professional fulfillment mean scores increased by 10.7% in the intervention group vs no change in the control group (absolute difference, 0.59 points; 95% CI, 0.01-1.16 points; *P* = .046). Work engagement increased by 6.3% in the coached group vs a decrease of 2.2% in the control group (absolute difference, 0.33 points; 95% CI, 0.02-0.65 points; *P* = .04). There were no statistically significant differences in mean scores for self-valuation, effect of work on personal relationships, or quality of life.

The cost of coaching per participant was calculated in 2 ways, which are listed in eTable 1 in Supplement 2. The first calculation included training in order to identify onboarding costs, and the second excluded training to consider ongoing costs. These costs were then compared with the cost listed per participant ($1400) in the Dyrbye et al^18^ coaching study; there was an onboarding cost increase of $156.73 and an ongoing cost savings of $430.77 per participant.

Coaching session themes reported by the intervention group participants are presented in Table 4. eTable 2 in Supplement 2 highlights comments about specific changes experienced, organized by theme. Fidelity assessment showed high frequency of excellent communication (43 [91.5%]), establishment of a coaching vision (41 [87.2%]), goal creation with progress made (46 [97.9%]), and coaches providing guidance rather than giving advice (45 [95.8%]). Finally, 100% of participants in the intervention group indicated that they would consider coaching in the future.

**Table 4.** Themes Addressed During Coaching Sessions (n = 47)

Theme	No. of participants (%)
Engaging in self-care	31 (66.0)
Integrating personal and professional life	30 (63.8)
Forging career paths aligned with personal and/or institutional values	28 (59.6)
Optimizing meaning in work	21 (44.7)
Improving work efficiency	21 (44.7)
Promoting self-advocacy and efficacy	24 (51.1)
Building leadership skills	18 (38.3)
Pursuing new opportunities	19 (40.4)
Pursuing hobbies and recreation	17 (36.2)
Addressing workload	20 (42.6)
Strengthening relationships outside of work	14 (29.8)
Building social support and community at work	13 (27.7)
Improving negotiation skills	11 (23.4)
Resolving interpersonal conflict	10 (21.3)

## Discussion

In this randomized clinical trial of an individualized coaching intervention for physicians by professionally trained peers, coached participants had statistically significant improvements in interpersonal disengagement, burnout, professional fulfillment, and work engagement compared with control group participants. These outcomes suggest that individualized coaching by physician peers may be an effective tool for improving physician well-being.

Many organizations may struggle to identify interventions to target the needs of their physicians and transmit value and appreciation from leadership. Based on the vast focus areas reported in the postcoaching assessment, coaching covered many areas important to physician well-being, and fidelity to the coaching intervention was high. The unanimous program satisfaction suggests that this intervention was appreciated and desired by participants.

To our knowledge, this study is the first physician coaching intervention to show a statistically significant improvement in workplace engagement in contrast to a control group whose engagement decreased over time. Higher physician engagement correlates with improved patient care, safety, and satisfaction; reduced health care costs; and higher physician satisfaction and retention.^32,33,34,35^ The statistically significant improvements in the PFI are an important finding, as the PFI has been shown to be associated with lower self-reported medical errors and sleep-related impairment, as well as lower burnout. Additionally, these findings show a possible mechanism for how an individually focused intervention delivered by an organization can lead to improvement in systems-based drivers of burnout, as themes learned from individual coaching were shared with institutional leadership and inspired additional initiatives.^36,37^ Additional studies to assess the effect of coaching on these outcomes are necessary.

Although self-valuation did not meet statistical significance thresholds between groups, it did increase in the coached group. Self-valuation involves the prioritization of personal well-being and a growth mindset perspective to learn and improve. Low self-valuation is associated with burnout.^28^ We expected to see an increase in self-valuation after coaching due to the focus on growth mindset and self-prioritization. As organizations seek to address burnout, the association of a coaching intervention with self-valuation should be noted.^38^

The authors of a recent study on surgeon coaching using external coaches tracked the persistence of their burnout reduction findings, noting that the effect of the intervention waned over the course of 6 months, with physicians returning to their preintervention baseline level of burnout.^20^ Assessment of the outcomes of well-being interventions over time is an important next step in understanding the value of such an investment.

It is important to note that no well-being intervention can stand alone in terms of its purported effect and reach. A coaching program is best suited in a portfolio of evidence-based interventions targeting individual- and systems-level drivers of well-being. Coaching programs offered by health care organizations may serve as individual- and systems-level supports. Coaching may improve the experience of coached individuals, who use these skills within the system in which they work. The use of internal coaches creates a cadre of individuals with unique training and expertise to communicate systems-level challenges identified in their coaching interactions with organizational leadership. For example, physician-coaches have also been involved in group coaching programs for physicians at high risk for burnout and retreats focused on organizational posttraumatic growth in a postpandemic era.^39,40^ When comparing costs of this program with previously published interventions using external coaches, after an initial investment, ongoing cost savings of more than $400 per physician coached were noted. Therefore, despite requiring an upfront investment, this intervention may provide a sustainable approach for an institution.

### Limitations

This study has several notable limitations. Clinicians participated voluntarily, which led to a group not entirely representative of the physician population within our organization and that may be more likely to have a positive response to the intervention. Specifically, women were overrepresented in our study, a phenomenon observed in other coaching studies that warrants further exploration.^20^ Additionally, our approach to enrollment on a voluntary, first-come, first-served basis selects for physicians who may have more free time or workplace engagement to respond to study announcements. Despite possible selection bias, every physician expressing interest during the enrollment period was able to enroll.

In analyzing the data, we adhered to a modified intention-to-treat approach, with a small number of initially randomized participants who did not receive coaching lost to follow-up. One could argue that engaging in coaching introduces bias of choice to the sample and weakens the effects of randomization.

Notably, we did not see changes in several of our outcome measures, including mMBI and effect of work on personal relationships. It is possible that this intervention delivered over a short period may not best address those components or may not be best measured by these tools given the time frequency response options. It is also possible that our sample size was not large enough to capture changes in these areas, as studies of a similar design have shown a reduction in overall burnout, emotional exhaustion, and quality of life.^19^

Participants randomized to the control group were aware of their opportunity to receive coaching upon completion of the initial 3 months. The expectation of future intervention may have provided a stabilizing effect for some of the factors measured in the control group.

Finally, a potential limitation when interpreting the results is the clinical importance of statistically significant differences in the measures of physician well-being. This study did not examine the longevity of these effects or what additional changes may be observed with the intervention over a longer time, which are aims for future studies.

## Conclusions

The findings from this randomized clinical trial suggest that training physicians professionally to be peer coaches may reduce interpersonal disengagement and burnout and improve professional fulfillment and work engagement for physicians. The potential for a long-term influence on hospital culture and physician retention warrants further exploration of peer coaching to add to the evidence base for addressing physician burnout.

## Notes

Supplement 1.
Trial Protocol and Detailed Institutional Review Board Protocol

Supplement 2. **eTable 1.** Cost of Coaching Per Participant **eTable 2.** Comments and Themes About Coaching Changes

Supplement 3.
Data Sharing Statement
