# Supplementary material for: Physician Coaching by Professionally Trained Peers for Burnout and Well-Being: A Randomized Clinical Trial
Source: JAMA Netw Open. 2024 Apr 12;7(4):e245645. doi: 10.1001/jamanetworkopen.2024.5645 (PMC11015346; doi:10.1001/jamanetworkopen.2024.5645)
Supplement: Supplement 1. — Trial Protocol and Detailed Institutional Review Board Protocol [file jamanetwopen-e245645-s001.pdf]

Protocol: Effect of Physician Coaching by Professionally Trained Peers on Burnout and Well-being: A Randomized Controlled Trial

Investigators: Kerri Palamara, MD; Stephanie B Kiser, MD, MPH; David Sterns, MD, MPH; Poi Ying Lai, MS; Nora Horick, MS

1. Goals and Objectives
   a) Goal: We aim to investigate the impact of a 3 month coaching intervention delivered by trained faculty peer coaches on burnout, professional fulfillment, resilience, and self-valuation in Massachusetts General Physician Organization (MGPO) faculty.
      i. Specific research question to be addressed and tested: Does coaching by certified peer coaches improve well-being, professional fulfillment, and self-valuation, and decrease burnout in faculty physicians compared to non-coached controls?
   b) Objectives:
      i. Create space for periodic reflection and goal-setting for MGPO faculty to visualize their career progress and trajectory.
      ii. Maximize the potential for continued professional development for MGPO faculty.
      iii. Evaluate whether professional development coaching by trained faculty peer coaches reduces burnout and improves professional fulfillment, resilience, and self-efficacy for MGPO faculty by reinforcing coping skills and strengths.
2. Methods

a) The MGPO coaching project is designed as a 9-month RCT enrolling faculty coachees at the beginning of the study, with crossover of the intervention and control groups at 3 months.

b) Study Subjects: The study population receiving coaching ("coachees") will be MGPO physicians. Coaches will be MGPO physician faculty certified as health and wellness coaches through the Wellcoaches Coach Training Program. Coachees will be recruited through two email announcements with embedded survey links to provide informed consent.

c) Sample size: The study plan is to enroll a minimum of 100 physicians. This will provide 80% power to detect a 0.3-0.5 SD minimally important difference (MID) effect size.[1]

d) Consent: all participants will provide electronic consent.

e) Randomization:

   i. All participants will be asked to provide demographic information (gender, age, ethnicity, marital status, clinical role, number of years in practice, faculty appointment, history of prior coaching, caregiving responsibilities) and complete an initial baseline wellness assessment (see components below in Surveys) via Research Electronic Data Capture (REDCap) browser-based software.

   ii. Coachees will be randomized in a 1:1 fashion to the control or intervention arm based on their initial baseline wellness assessment. Randomization will be stratified by gender (woman, man, non-binary, or prefer not to say) and department of practice (Department of Medicine or other).

   iii. Those randomized to control will be offered standard institutional resources that currently exist for burnout and wellness.

iv. Those randomized to intervention will be assigned a faculty coach for a 3-month period. Coaches will be assigned on a first come, first serve basis, based on coachee preference and coach availability. Coaches have previously been certified as health and wellness coaches through Wellcoaches.

v. Coaches and coachees will be responsible for meeting together for a total of 6 coaching sessions over a 3-month period, a 1.5-hour initial coaching session followed by 5 additional 60-minute sessions. The coaching sessions will follow the Wellcoaches training model with the initial coaching session including facilitated introductions and rapport building, setting expectations, assessment of coachee strengths and creation of a wellness vision, three-month and weekly goals. The focus for each follow-up coaching session will include a check-in, goal review, generative moment, goal setting, and session conclusion.

vi. After 3 months, coaching will end for participants assigned to the intervention group. Participants in the control group when then be assigned to coaches and complete a 6 session intervention over the course of 3 months as described above.

vii. Schematic:

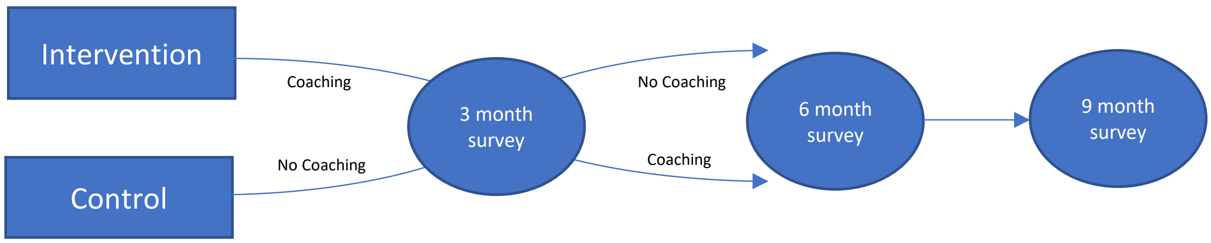

f) Surveys: All participants will be asked to complete electronic surveys at baseline, 3 months., 6 months and 9 months. Validated instruments will be used to measure burnout,

professional fulfillment, well-being and self-valuation. Surveys will be administered electronically by the MGH study team. We will use the modified Maslach Burnout Inventory (mMBI), the Stanford Professional Fulfillment Index (PFI), the Utrecht Work Engagement Scale-9 (UWES-9), the Self-Valuation Scale (SVS), the Quality of Life (QoLS) and Impact of Work on Personal Relationships (IWPR) Scales.[2-11]

g) Data handling: Surveys will be administered via REDCap and results will be downloaded to the MGH study team. Study participants will have their sequential survey results linked by a unique identifier known only to the statistical analyst and destroyed after collection of all data. Therefore, data will be deidentified for all study personnel.

h) Data analysis

   i. Descriptive statistics for demographic variables, intervention v control.

   ii. Score scaled items to create composite variables for key outcomes (mMBI, PFI, UWES-9, SVS, QoLS and IWPR) using total scores and subscale scores as appropriate, and following guidelines for using scores as continuous or categorical cut points according to manuals provided by scale developers.[1–7]

   iii. Compare key outcomes in change over time between intervention and control groups.

References

1. Farivar SS, Liu H, Hays RD. Half standard deviation estimate of the minimally important difference in HRQOL scores? *Expert Rev Pharmacoecon Outcomes Res*. 2004;4(5):515-523. doi:10.1586/14737167.4.5.515

2. Maslach C, Jackson SE. The measurement of experienced burnout. *J Organ Behav*. 1981;2(2):99-113. doi:10.1002/job.4030020205

3. West CP, Dyrbye LN, Sloan JA, Shanafelt TD. Single Item Measures of Emotional Exhaustion and Depersonalization Are Useful for Assessing Burnout in Medical Professionals. *J Gen Intern Med*. 2009;24(12):1318-1321. doi:10.1007/s11606-009-1129-z

4. Trockel M, Bohman B, Lesure E, et al. A Brief Instrument to Assess Both Burnout and Professional Fulfillment in Physicians: Reliability and Validity, Including Correlation with Self-Reported Medical Errors, in a Sample of Resident and Practicing Physicians. *Acad Psychiatry*. 2018;42(1):11-24. doi:10.1007/s40596-017-0849-3

5. Trockel MT, Menon NK, Rowe SG, et al. Assessment of Physician Sleep and Wellness, Burnout, and Clinically Significant Medical Errors. *JAMA Netw Open*. 2020;3(12):e2028111. doi:10.1001/jamanetworkopen.2020.28111

6. Wilmar Schaufeli, Arnold Bakker. Utrecht Work Engagement Scale: Preliminary Manual (Version 1.1). Published online 2004. Accessed December 21, 2021. https://www.wilmarschaufeli.nl/publications/Schaufeli/Test%20Manuals/Test_manual_UWES_English.pdf

7. Trockel MT, Hamidi MS, Menon NK, et al. Self-valuation. *Mayo Clin Proc*. 2019;94(10):2022-2031. doi:10.1016/j.mayocp.2019.04.040

8. Boer AGEM de, Lanschot JJB van, Stalmeier PFM, et al. Is a Single-Item Visual Analogue Scale as Valid, Reliable and Responsive as Multi-Item Scales in Measuring Quality of Life? *Qual Life Res*. 2004;13(2):311-320.

9. Trockel J, Bohman B, Wang H, Cooper W, Welle D, Shanafelt TD. Assessment of the Relationship Between an Adverse Impact of Work on Physicians’ Personal Relationships and Unsolicited Patient Complaints. *Mayo Clin Proc*. 2022;97(9):1680-1691. doi:10.1016/j.mayocp.2022.03.005

10. Buchbinder SB, Wilson M, Melick CF, Powe NR. Primary care physician job satisfaction and turnover. *Am J Manag Care*. 2001;7(7):701-713.

11. Hann M, Reeves D, Sibbald B. Relationships between job satisfaction, intentions to leave family practice and actually leaving among family physicians in England. *Eur J Public Health*. 2011;21(4):499-503. doi:10.1093/eurpub/ckq005

PARTNERS HUMAN RESEARCH COMMITTEE
DETAILED PROTOCOL

Please submit a protocol that includes all of the following sections. You may submit a sponsor's or other pre-existing protocol, **however a grant application is not considered a protocol.** Protocols must include a version number and/or date and all pages must be numbered.

Version: 4
Date: 05/08/2022

I. BACKGROUND AND SIGNIFICANCE (including progress report and preliminary studies).

a. Historical background
b. Previous pre-clinical or clinical studies leading up to, and supporting the proposed research
c. Rationale behind the proposed research, and potential benefits to patients and/or society

Coaching is used in business and many other career paths to help the individual define and create their own goals and strategies for achieving those goals. The MGH Professional Development Coaching Program applies this model to help our residents in their professional development as physicians. This has been successful in providing our housestaff with the opportunity for a non-program director faculty member to review their entire portfolio and development throughout residency in a coaching role. It was successfully developed and rolled out in 2011 using novice faculty coaches trained to use positive psychology and coaching skills with a trainee.[1] This program was evaluated from 2012-present, and based on its success, it has since been adopted by >25 internal medicine residency programs around the country. In 2017 we began to investigate the impact of coaching compared to non-coached peers in a randomized trial among non-internal medicine residency programs and internal medicine subspecialty fellowship to understand the impact of this program and its generalizability. Data from all of these studies has suggested that coaching is effective in allowing trainees to understand their development over time, find meaning and purpose in their work, and identify their strengths and how to use these to overcome challenges and stressors. Additionally, there is a benefit to the coaches themselves, who are able to connect with other faculty coaches in a rewarding way, that provides faculty development in leadership development and positive psychology, and space to interact with a group of like-minded physicians.[2]

From the work we have done with housestaff through the MGH Professional Development Coaching Program we have seen a tremendous interest from faculty members for access to similar services. Prior studies show improvement in faculty burnout and engagement at work through small-group sessions focused on reducing distress and promoting well-being.[3] We have also seen that while the training of novice coaches in positive psychology is sufficient to begin crucial conversations about drivers of well-being, the need for more in-depth coaching with certified coaches exists.

The goal of this project is to expand coaching to MGH faculty members and Advance Practice Providers (APPs) at MGH and also provide more in-depth training for coaches through the International Federation of Coaching, through the Wellcoaches Coach Training Program. This is a unique approach to professional development within the field of medicine that has not yet been employed or studied. There was a recent publication of professional coaches hired outside of the field for faculty development, but to our knowledge there has been no training of medical colleagues with professional coaching skills. This has the potential to provide new data for the field and also become a sustainable intervention for MGH in addressing ongoing professional development for our faculty, our Advanced Practice Providers, and the burnout epidemic. Finally, this can serve as model for implementation in other institutions.

II. **SPECIFIC AIMS** (Research Objectives)

a. Specify objectives and hypotheses to be tested in the research project

Objectives:

1. Create space for periodic reflection and goal-setting for MGPO faculty and MGH APPs to visualize their career progress and trajectory.

2. Provide an avenue for MGPO faculty and MGH APPs to be trained as professional peer coaches.

3. Maximize the potential for continued professional development for MGPO faculty and MGH APPs.

4. Improve burnout, professional fulfillment, resilience, and self-efficacy for faculty and MGH APPs by reinforcing coping skills and strengths.

5. Evaluate whether professional development coaching by trained faculty peer coaches reduces burnout, improves emotional well-being and improves professional fulfillment in MGPO faculty coaches and MGH APPs.

Hypothesis: MGPO-Faculty and MGH APPs will benefit from a Professional Development Coaching Program in the following ways:

1. A Professional Development Coaching Program, will improve faculty and APP well-being, compared to non-coached controls.

2. A Professional Development Coaching Program will improve faculty and APP workplace satisfaction, compared to non-coached controls.

3. 3. A Professional Development Coaching Program will decrease burnout for faculty and APP participants and faculty coaches and improve their resiliency by allowing them to visualize their accomplishments, improve their response to stress of uncertainty, and decrease emotional exhaustion, compared to controls.

4. 4. A Professional Development Coaching Program will improve faculty coach well-being, compared to non-coach controls.

III. **SUBJECT SELECTION**

a. Inclusion/exclusion criteria

Inclusion criteria for faculty participants to receive coaching: faculty within the Mass General Physicians Organization (MGPO), and APPs practicing within Massachusetts General Hospital including at the Boston main campus and satellite community sites, no specific age range, screening based on self-referral through email

Inclusion criteria for faculty participants to become a coach: faculty within the MGPO, no specific age range, screening based on self-referral and information provided in a standard application assessing motivation to become a faculty coach

b. Source of subjects and recruitment methods

MGPO-Faculty and MGH APPs will be sent an email to make them aware of the program for voluntary enrollment. It will be clearly described that their participation is not mandatory.

IV. SUBJECT ENROLLMENT

a. Methods of enrollment, including procedures for patient registration and/or randomization

MGPO-Faculty and MGHAPPs will be sent an email to make them aware of the program for voluntary enrollment, including both the opportunity to receive coaching in the trial or to become a trained coach. These surveys will serve no evaluative purpose in their job performance. It will be clearly described that their participation is not mandatory. Participants interested in receiving coaching will be placed into 2 groups via simple randomization. Randomization will be carefully done to ensure gender and department are balanced between the groups. Participants interested in becoming a trained coach will fill out a short application of interest from which 15 individuals will be selected to participate in coach training.

b. Procedures for obtaining informed consent (including timing of consent process)

Participants will be invited via email as described above. Details of the study will be included in the email. Electronic consent will be obtained upon declaring interest in the program, as well as in emails when surveys are sent out.

c. Treatment assignment, and randomization (if applicable)

Randomization will place participants interested in receiving coaching in 2 groups, either coaching for 3 months, or usual mentoring and well-being practices that exist in their department and through MGH. Randomization will be carefully done to ensure gender and faculty department are balanced between the groups. At the 3 month mark these groups will switch and participants that were randomized to usual mentoring will be assigned a trained faculty coach for an additional 3 months. Participants will have access to the biographies of trained coaches after being assigned to receive coaching in their respective groups and will be able to select a coach they feel best matches their needs pending coach availability.

V. STUDY PROCEDURES

a. Study visits and parameters to be measured (e.g., laboratory tests, x-rays, and other testing)

Participants will be enrolled and followed with data collection for 9 months. 3 months of that time will be spent with a professional development coach with a meeting lasting 30 minutes to 1 hour every 2 weeks. All data collection will be done via survey online at the specified time point. Each coaching session will occur over a secure media platform by Bongomedia. This platform has been adopted by leadership at MGH and will provide deidentified, anonymized contect of the coaching sessions for program leadership. Coaches and participants being coached will have the opportunity to opt out of use of this platform.

b. Drugs to be used (dose, method, schedule of administration, dose modifications, toxicities), include Toxicity Grading Scale (if applicable)

N/A

c. Devices to be used

N/A

d. Procedures/surgical interventions, etc.

N/A

e. Data to be collected and when the data is to be collected

Several previously validated survey tools will be utilized for data collection including the Professional Fulfillment Index, the Self Valuation Scale, the Ultrecht Work Engagement Scale, Abbreviated Maslach Burnout Inventory and a Negative Impact of Work on Relationships Scale. Data will be collected at enrollment, 3 months, 6 months and 9 months. All surveys will be filled out by both participants enrolled and randomized for coaching and by trained faculty coaches. These surveys will be sent at enrollment, 3 months, 6 months and 9 months. In addition, both groups will fill out an intervention assessment after sessions that will gather data on themes addressed in the sessions and how effective the sessions felt to the participant. Additionally, all sessions conducted on the Bongomedia platform will produce deidentified, anonymized content that will be aggregated for review.

VI. BIOSTATISTICAL ANALYSIS

a. Specific data variables being collected for the study (e.g., data collection sheets).

Demographic data including gender, number of years as faculty and department will be collected. Survey data will be collected based on scales previously noted.

b. Study endpoints

We will survey the faculty and APP participants with an assessment of the Professional Development Coaching Program at enrollment, 3 months, 6 months and 9 months. After the 9 month survey the study will be completed.

c. Statistical methods

Assistance with statistical data analysis will be requested through the Department of Medicine. We will use two-sample t-tests or Wilcoxon rank sum test, whichever more appropriate to compare between groups for the changes from baseline at the different time points for all metrics between the faculty coaches and faculty participants. We will include all subjects in the primary comparisons and explore subgroup analysis stratified by participant characteristics (sex, sub-specialty department)

d. Power analysis (e.g., sample size, evaluable subjects, etc.)

N/A

VII.. RISKS AND DISCOMFORTS (Stratify by common and uncommon)

a. Complications of surgical and non-surgical procedures, etc.

N/A

b. Drug side effects and toxicities

N/A

c. Device complications/malfunctions

N/A

d. Psychosocial (non-medical) risks

The usual potential harms that these physicians or APPs may encounter in the course of their work in patient care are expected and their health and safety will be safeguarded as per the usual hospital policies at MGH. All participants interested in receiving coaching will be randomized but coaching program and survey participation is voluntary and all will have access to coaching at some time point during their enrollment mitigating the effect of potential harm by not receiving coaching. In the study there is risk of discomfort for the faculty participants and coaches in filling out surveys about burnout and their program experience. This risk will be minimized by

surveys which are de-identified and only viewed by research staff not affiliated with their department. There will be no consequence if participants do not complete the survey and this will be explained before completing the survey.

Confidentiality will be required of the coaches. Specifically, they will be required not to share the details of confidential evaluations with other faculty. Coaches are not asked to report back on their meetings to their department or the participant's department, unless they are concerned about the safety and well-being of the faculty or APP participant, patients, or others. The usual potential harms that these physicians and APPs may encounter in the course of their work in patient care are expected and their health and safety will be safeguarded as per the usual hospital policies at MGH. Since there are no treatment plans or procedures involved, there is no risk to the safety of the subjects. Additionally, all faculty trained to be a coach will undergo specific training developed by the institutional Employee Assistance Program, Human Resources and the Office of General Council.

e. Radiation Risks (statement provided by Radiation Safety Committee)
N/A

VIII. POTENTIAL BENEFITS

a. Potential benefits to participating individuals

All faculty and APP study participants in the Professional Development Coaching Program are expected to receive the same benefit, regardless of participation in the surveys or the time point at which they randomized to professional coaching; which is increased awareness of their accomplishments, increased opportunity for reflection, decreased burnout, improved well-being, increased resiliency, and increased opportunity to set self-directed learning goals. Additional benefits which are likely, but may be harder to capture in a survey, are increased awareness of where to turn in the event that they are struggling with work, life or professional issues.

b. Potential benefits to society (e.g., increased understanding of disease process, etc.)

This is a unique approach to professional development within the field of medicine that has not yet been employed or studied. There was a recent publication of professional coaches hired outside of the field for faculty development, but to our knowledge there has been no training of medical colleagues with professional coaching skills. This has the potential to provide new data for the field and also become a sustainable intervention for MGH in addressing ongoing professional development for our faculty and Advanced Practice Providers and the burnout epidemic. Finally, this can serve as model for implementation in other institutions.

IX. MONITORING AND QUALITY ASSURANCE

a. Independent monitoring of source data

Survey data will be reviewed monthly using online, de-identified collection via RedCap. The data will be reviewed by study researcher within the MGH Department of Medicine. Anonymous, de-identified data will then be reviewed by study investigators. All data will be stored in a secure Partners electronic folder protected by a password on a network drive.

b. Safety monitoring (e.g., Data Safety Monitoring Board, etc.)
N/A

c. Outcomes monitoring

Outcomes will be evaluated at the end of data collection.

d. Adverse event reporting guidelines

No significant reportable events anticipated. If this happens in an unanticipated fashion we will notify the IRB immediately.

X. REFERENCES

1. Palamara K, Kauffman C, Stone V, Bazari H, Donelan K. Promoting Success: A Professional Development Coaching Program for Interns in Medicine. JGME. 2015;7(4):630-7.
2. Palamara K, Kauffman C, Chang Y, Barreto EA, Yu L, Bazari H, Donelan K. Professional Development Coaching for Residents: Results of a 3-Year Positive Psychology Coaching Intervention. J Gen Intern Med. 2018 11; 33(11):1842-1844.
3. West CP, Dyrbye LN, Rabatin JT, Call TG, Davidson JH, Multari A, et al. Intervention to Promote Physician Well-being, Job Satisfaction, and Professionalism. JAMA Intern Med. 2014;174(4):527.
