# Supplementary material for: Physician Coaching by Professionally Trained Peers for Burnout and Well-Being: A Randomized Clinical Trial
Source: JAMA Netw Open. 2024 Apr 12;7(4):e245645. doi: 10.1001/jamanetworkopen.2024.5645 (PMC11015346; doi:10.1001/jamanetworkopen.2024.5645)
Supplement: Supplement 2. — eTable 1. Cost of Coaching Per Participant eTable 2. Comments and Themes About Coaching Changes [file jamanetwopen-e245645-s002.pdf]

Supplemental Online Content

Kiser SB, Sterns JD, Lai PY, Horick NK, Palamara K. Physician coaching by professionally trained peers for burnout and well-being: a randomized clinical trial. *JAMA Netw Open*. 2024;7(4):e245645. doi:10.1001/jamanetworkopen.2024.5645

eTable 1. Cost of Coaching Per Participant

eTable 2. Comments and Themes About Coaching Changes

eReference

This supplemental material has been provided by the authors to give readers additional information about their work.

© 2024 Kiser SB et al. *JAMA Network Open.*

eTable 1. Cost of Coaching Per Participant

	Cost	Notes
Coach Training (13 coaches at $2350 each)	$30,550	Wellcoaches Cost
Coach Payments (252 coaching hours at $200/hr)	$50,400	
Total Cost	$80,950	
Cost per participant (52) including training	$1,556.73	Onboarding cost
Cost per participant (52) excluding training	$969.23	Ongoing cost
Cost of Mayo study per participant[1]	$1,400	
Cost differential onboarding	($156.73)	
Cost differential ongoing	$430.77	

© 2024 Kiser SB et al. *JAMA Network Open.*

eTable 2. Comments and Themes About Coaching Changes

© 2024 Kiser SB et al. *JAMA Network Open.*

What changes did you make as a result of coaching?	Theme
Think about things in a more objective organized manner	Addressing workload
Less angry and overwhelmed	Addressing workload
I felt much more encouraged about my work. I set goals and achieved them. I felt more optimistic about my work, more in line with how it should be.	Addressing workload
I have made many structural changes to my day to foster focused goal-oriented work. I also made changes to how I receive notifications, and to how I alert patients to results, coordinate follow-up, etc.	Addressing workload
Considered my leadership strengths	Building leadership skills
Considered my goals for leadership	Building leadership skills
Strategizing	Building leadership skills
Better planning before difficult conversations	Building leadership skills
Delegating more to support staff	Building leadership skills
Use my voice Believe in what I have to offer Be clear in agenda setting with meetings View meetings as information gathering rather than judgements	Building leadership skills
increased proactive communication with colleagues and mentors.	Building leadership skills
Clarify leadership style, re-structure team structure and reset priorities	Building leadership skills
More awareness of my personality having strengths, rather than weaknesses. Accepting my introversive personality works best 1:1, rather than dominating large group meetings. Dealing with meeting agendas before and after the meeting. Previously worried that I am unable to make decisions for fear of being wrong. Now, accepting that listening to others before making a decision does not always show weakness and indecisiveness.	Building leadership skills
I focused on the environments that I do have the ability to make psychological safety for those that I work with. Instead of being afraid and siloing myself off I have made an effort to check in with friends and colleagues to both support them and to realize the support that I do have. I do not say that I am fine when I am not fine.	Building social support and community at work
No major change; just an internal understanding that I am actually happy with the status quo and don't need or desire change.	Building social support and community at work
Integrating mindfulness to daily life	Engaging in self-care
Improved weight and sleep	Engaging in self-care
Stress reduction	Engaging in self-care
increased self care	Engaging in self-care
Thinking longer term for priorities and goals	Forging career paths aligned with personal and/or institutional values
I got more concrete about my various plans and goals	Forging career paths aligned with personal and/or institutional values
Dedicated "focus time" multiple times/week	Improving work efficiency
I learned to say "no" and prioritize my tasks.	Improving work efficiency

© 2024 Kiser SB et al. *JAMA Network Open.*

I am much more aware of my perfectionist tendencies and able to acknowledge them and put them aside more than I was able to before thereby increasing my efficiency at work; changed my perspective on public speaking and have become less anxious about it as a result; gained perspective on my feelings of impostor syndrome and am starting to feel more confident in work situations as a result.	Improving work efficiency
Made note templates to speed note writing	Improving work efficiency
Note writing efficiency	Improving work efficiency
Implemented some strategies to reduce uncertainty when I was delivering clinical care, like previewing caseload for the next day and working on treatment plan ahead of time	Improving work efficiency
Looking ever more closely at reducing my time at work and pursuing other, more rewarding, activities.	Integrating personal and professional life
Changed my morning routine to balance personal needs and preparation for the work day. Prioritized pre-charting every morning	Integrating personal and professional life
Shut off work more when with family	Integrating personal and professional life
Better work life balance	Integrating personal and professional life
Improved setting boundaries/limits with my time related to work	Integrating personal and professional life
Thinking about the meaning of work and goals	Optimizing meaning in work
I am giving up some responsibilities to focus on other things that I find more fulfilling in my work and to hopefully have less stress.	Optimizing meaning in work
More positive thinking	Optimizing meaning in work
I was able to slow down a bit and prioritize some things, as well as making specific plans to tackle some issues like making time to work on projects with "soft" deadlines.	Promoting self-advocacy and efficacy
Let go about guilt about career path, made a dedicated plan for self-education next year, made a plan for improving as a medical educator, more self-reflection and honesty	Promoting self-advocacy and efficacy
Took some concrete steps on some projects I'd been behind on. Made some changes in my work schedule to better reflect my professional aspirations.	Promoting self-advocacy and efficacy
Reduced sessions at work. Changed template to allow time for urgent tasks during work days.	Promoting self-advocacy and efficacy
Small changes to ensure that I do regular things to take care of my health, feel good about my accomplishments, have quality moments with my family.	Promoting self-advocacy and efficacy
I blocked time from my schedule one morning a week and started taking art lessons during that time to help reduce burnout and isolation I was experiencing with working from home. I addressed an issue with a colleague that had been bothering me.	Promoting self-advocacy and efficacy

© 2024 Kiser SB et al. *JAMA Network Open.*

I am engaging in more mindfulness, taking time for myself and setting boundaries with others. I am more able to protect my "me" time.	Promoting self-advocacy and efficacy
Being better at recognizing my worth and what I am able to accomplish	Promoting self-advocacy and efficacy
Setting realistic goals	Promoting self-advocacy and efficacy
More self-compassion. Modifying negative thought patterns. Openness of communication and vulnerability.	Promoting self-advocacy and efficacy
Improved approach/mindfulness around self-critique.	Promoting self-advocacy and efficacy
I felt more confident and felt like she provided strategies to make me cope better with my own sense of insecurity.	Promoting self-advocacy and efficacy
More self compassion and introspection	Promoting self-advocacy and efficacy
I am definitely working to advocate for myself. Before this experience, it was difficult for me to put my finger on why I felt somewhat stagnant and approaching burnout in my career. Now I have a clearer picture of what I want and also a framework for how to make future career choices.	Promoting self-advocacy and efficacy
I began to set goals related to promotion while also exploring opportunities to transition to a new location. I felt that goal-setting and reframing my thinking towards a more positive/goal-directed way helped with many things that were causing cognitive burden/load including how disorganized some things have been at work (outside of my control).	Promoting self-advocacy and efficacy
I had more clarity of my goals and am pursuing new job avenues.	Pursuing new opportunities
Felt more open-ness to exploring roles outside of the instutution	Pursuing new opportunities
Listening to my "inner voice" as I am interacting with people in difficult situations.	Resolving interpersonal conflict
More effort to build personal friendships outside of work	Strengthening relationships outside of work

eReference

1. Dyrbye LN, Shanafelt TD, Gill PR, Satele DV, West CP. Effect of a professional coaching intervention on the well-being and distress of physicians: a pilot randomized clinical trial. *JAMA Intern Med*. 2019;179(10):1406-1414. Medline:31380892 doi:10.1001/jamainternmed.2019.2425

© 2024 Kiser SB et al. *JAMA Network Open.*
