# Supplementary material for: Physician Coaching by Professionally Trained Peers for Burnout and Well-Being: A Randomized Clinical Trial
Source: JAMA Netw Open. 2024 Apr 12;7(4):e245645. doi: 10.1001/jamanetworkopen.2024.5645 (PMC11015346; doi:10.1001/jamanetworkopen.2024.5645)
Supplement: Supplement 3. — Data Sharing Statement [file jamanetwopen-e245645-s003.pdf]

Data Sharing Statement

Kiser. Physician Coaching by Professionally Trained Peers for Burnout and Well-Being. *JAMA Netw Open*. Published April 12, 2024. doi:10.1001/jamanetworkopen.2024.5645

Data
Data available: No
